# Molecular, Physical, and Technical Performance Response After a Competitive Match in Male Professional Soccer Players

**DOI:** 10.3390/antiox14010073

**Published:** 2025-01-09

**Authors:** Alessandra Modesti, Rosamaria Militello, Alice Tanturli, Alice Santi, Massimo Gulisano, Cristian Petri, Luca Pengue, Alessio Pellegrino, Pietro Amedeo Modesti, Simone Luti

**Affiliations:** 1 Department of Experimental and Clinical Biomedical Sciences, University of Florence, 50134 Florence, Italy; alessandra.modesti@unifi.it (A.M.); rosamaria.militello@unifi.it (R.M.); alice.santi@unifi.it (A.S.); 2 Department of Experimental and Clinical Medicine, University of Florence, 50134 Florence, Italy; alice.tanturli@unifi.it (A.T.); massimo.gulisano@unifi.it (M.G.); alessio.pellegrino@unifi.it (A.P.); pa.modesti@unifi.it (P.A.M.); 3 A.C.F. Fiorentina S.r.l., 50137 Florence, Italy

**Keywords:** oxidative stress, antioxidant potential, IgA, metabolomics, recovery, GPS technology, plasma, soccer

## Abstract

Background: Male professional soccer players frequently compete in multiple matches weekly, and each match significantly impacts their homeostasis, health, and performance. This study evaluates players response at 48 h post-match by combining biological and GPS data. Investigating biochemical and performance metrics offers insights into the physical demands of high-intensity exercise, essential for optimizing performance, recovery, and overall athlete health. Methods: The study involved an Italian “Serie A” team, and we assessed players’ effort during a single match using GPS data and compared it to “Serie A” averages. Additionally, we evaluated oxidative stress and metabolism 48 h after the match. Results: At 48 h post-match, there were no signs of oxidative stress and changes in salivary IgA levels, but total antioxidant potential was significantly low. Moreover, increased plasma metabolites linked to energy production were also observed. Conclusions: The results indicate that 48 h after a match in “Serie A”, well-trained athletes showed no oxidative stress, to the detriment of the antioxidant potential, along with increased metabolites crucial for energy production. Combining GPS and metabolic analysis enhances player performance, informs tactical decisions, and supports team success, fostering data-driven approaches in soccer.

## 1. Introduction

In recent decades, the metabolic demands of soccer performance have been extensively studied and it is known that 90 min of a football match induces a modification in the body homeostasis of athletes [1,2,3]. It is also known that these alterations affect many physiological processes in the hours and days following the match [4,5].

The evaluation of single molecules for use as markers of fatigue cannot identify the complex processes that underlie recovery from exercise. On the contrary, a multi-molecules panel and individual analysis could provide a robust assessment of player fatigue during recovery. The application of “sportomics” allows a global and non-invasive characterization of the biochemical changes that occur in response to exercise. It is an integrated approach that includes the simultaneous analysis of numerous metabolites through the use of analytical techniques in different biological samples with data processing methods to study metabolic changes induced by physical exercise.

We previously conducted metabolomic analyses on plasma and saliva samples to characterize the metabolic impact of training programs in both male and female elite football and basketball players. Our results highlighted that soccer players are subject to higher continuous oxidative stress, associated with possible inflammation, in comparison to basketball athletes. We found a lower plasma level in adiponectin in football athletes, and we suggest that this could be responsible for the local inflammatory effects of exercise loads [6]. In male and female basketball, we observed a decrease in cytoskeletal protein, involved in muscle regeneration, and Dermcidin, a stress-induced myokine linked to inflammatory events [7]. Moreover, we reported that trained women have greater antioxidant capacity than sedentary women, differences that we suggested are associated with an increase in plasma lactate level. Lactate is a regulatory molecule, a “lactormone” [8]. In previous experimental conditions, we found high levels of lactate and low salivary cortisol, leading to the conclusion that the type of training in basketball induces adaptation in well-trained athletes avoiding overtraining/overreaching [9]. In a recent paper we suggested that females and males have several common traits of adaptation to training. They both showed an increase in plasma antioxidant potential and lactate plasma level, as well as an activation of the detoxifying process [10].

By understanding and effectively integrating acute exercise sessions such match-play during a training period, athletes can maximize their performance and minimize the risk of injury and overtraining [5,11].

Global Positioning System (GPS) technology has been utilized to quantify positional profiles and temporal variations during match play. Given the challenges associated with directly measuring the exact energy cost of speed changes, a metabolic power calculation based on a theoretical model has been employed to estimate the energy expenditure associated with acceleration and deceleration in team sports. Young et al. [12] analyzed the metabolic power of hurling match-play, observing positional differences in metabolic power among different positions, suggesting that the integration of both metabolic power and GPS time-motion analysis metrics in hurling is recommended. Integrating GPS technology with metabolic analysis provides a powerful tool for optimizing player performance, improving tactical decisions, and enhancing overall team success [13,14,15]. GPS technology is used to monitor and analyze various aspects of player performance. It tracks the total distance a player covers during a game or training session and measures how fast a player runs and how they accelerate and decelerate. They assess the physical external load a player experiences, helping to manage training intensity and reduce the risk of injury.

Metabolism in the context of soccer refers to the biochemical processes that provide energy for physical activity. Understanding a player’s metabolism is crucial for optimizing performance and recovery [16].

The aims of this study are: (i) to understand and effectively integrate acute exercise sessions into a long training period, (ii) to integrate GPS data with metabolic analysis, (iii) to monitor the athletes’ response to acute exercise sessions (a single play match) throughout a long training period in order to understand how a single exercise session (acute exercise) interacts with long-term training program. Studying the effect of a single soccer match is crucial for optimizing player performance, ensuring effective recovery, preventing injuries, and informing strategic and tactical decisions. This comprehensive understanding helps in creating a holistic approach to training and match preparation, ultimately leading to improved team performance and individual player development.

## 2. Materials and Methods

### 2.1. Participants and Study Plan

A professional male soccer team from Italian Seria A (top league) participated in this study. The team trained 5 days per week. Training sessions included technical and tactical drills and physical training typical of an elite soccer team. Common recovery strategies included sleep, hydration, nutrition, mental recovery, active recovery, foam-rolling/massage, stretching, and cold-water immersion. All the players involved in this study adhered to a Mediterranean diet as reported by Petri et al. [17], without the addition of specific antioxidants during the period of the analysis.

All participants were fully informed about the procedures, methods, potential benefits, and risks associated with the study. The study adhered to the ethical standards for human experimentation outlined in the Declaration of Helsinki (1964) and its update in Fortaleza (2013). Each participant provided written informed consent to participate in the study, which received approval from the Ethics Committee of the University of Florence, Italy (AM_Gsport 15840/CAM_BIO). All measurements were conducted under resting conditions by the same operator to ensure consistency. Biological samples (plasma and saliva) were collected during the season 24 h before (now called “pre-match”) and 48 h after (now called “post-match”) an official competition of Italian Seria A. The main inclusion criterion was having played at least 60 min of the match. A total of 7 players were enrolled: 2 central defenders, 2 side defenders, 2 midfielders, and 1 striker.

Saliva samples (1 mL) were collected in the morning between 9:00 and 11:00 a.m. to minimize variations caused by circadian rhythms. Participants were instructed to abstain from eating, drinking, or brushing their teeth for at least 30 min prior to sample collection. Saliva was collected from each participant using Salivette Cortisol devices (Sarstedt AG & Co., Nümbrecht, Germany) and stored at −80 °C until analysis. The cotton swab from the Salivette was processed as described by Luti et al. [18].

A capillary blood sample was picked up using a heparinized Microvette CB300 (Sarstedt AG & Co.) from each participant as reported in Militello et al. [6]. Briefly, the blood sample was collected with a capillary tip, holding the Microvette in a horizontal position (filling volume 300 μL). To obtain plasma, the blood tubes were immediately centrifuged for ten minutes at 2000× *g* using a table centrifuge. Plasma samples were stored at −80 °C in a freezer and measurements of oxidative stress and metabolites were performed on stored samples. The reduced invasiveness, the simplicity of execution, and the lower cost led us to prefer the capillary collection to venous; moreover, small volumes of samples were sufficient to carry out all experiments.

### 2.2. Global Positioning System (GPS) Measurement

Movement data were collected using portable GPS units (K-sport k 50 wearable—Montelabbate, Pesaro-Urbino, Italy), the world’s smallest professional device with the highest sampling rate (50 Hz) [19].

The GPS unit was fitted into a custom pouch in the players’ guernsey, located between the scapulae. All players wore the same unit for each game during the season to minimize inter-unit error. The GPS metrics analyzed were adopted from previous research in professional Italian football teams to permit comparisons and include Total Distance (m, TD), Average Speed (m min^−1^, AS), High-Speed Running distance (16–20 km h^−1^, HSR), Maximum Speed Running distance (20–25 km h^−1^, MSR), Sprint Running distance (<25 km h^−1^, SR), and Low Speed Running distance (<11 km h^−1^, LSR) [20,21,22].

Other important values to describe the intensity of a performance are acceleration and metabolic power (MP). The team utilized the 4-2-3-1 formation for the match.

### 2.3. Plasma Oxidative Stress Measurements

The d-ROMs test (diacron Reactive Oxygen Metabolites) and the BAP Test (Biological Antioxidant Potential) were used to determine the levels of reactive oxygen metabolites and the antioxidant capacity on plasma as reported by Pinto et al. [10]. The biomarkers d-ROMs and BAP were selected based on their long-term stability. All analyses were carried out using a free radical analyzer system (free carpe diem—Diacron International srl) which included a spectrophotometric device reader and a thermostatically regulated minicentrifuge, and the measurement kits were optimized to the FREE Carpe Diem System, according to the manufacturer’s instructions.

### 2.4. Plasma Protein Carbonylation Analysis

Plasma samples of 20 µg were added to 4 × Laemmli buffer (0.5 M TrisHCl pH 6.8, 10% SDS, 20% glycerol, β-mercaptoethanol, and 0.1% bromophenol 45 blue), boiled for 5 min, and separated on 12% SDS/PAGE. Proteins were then transferred onto a PVDF membrane using the Trans-blot Turbo Transfer System (Bio-Rad Laboratories, Hercules, CA, USA). Protein carbonyls were derivatized to DNP by incubating PVDF membranes in 10 mM DNPH dissolved in 2 N HCl, for 1 h at room temperature. After washing the membranes three times with methanol, they were incubated overnight at 4 °C with the primary antibody 1:10,000 anti-DNP IgG antibody (Sigma, St. Louis, MO, USA) in Phosphate-buffered saline (PBS) containing 5.0% non-fat dry milk.

The blots were then washed with PBS, 0.1% (*v*/*v*) Tween, and incubated with the goat anti-rabbit IgG/HRP conjugate for 1 h at room temperature. Immune complexes were detected with the enhanced chemiluminescence (ECL) detection system (GE Healthcare, Chicago, IL, USA) and by an Amersham Imager 600 (GE Healthcare). The intensity of the immunostained bands was normalized with the total protein intensities measured by Coomassie brilliant blue R-250 from the same PVDF membrane.

The densiometric analysis was carried on using the ImageJ 1.53 program [23].

### 2.5. Salivary IgA Western Blot Analysis

Protein content in saliva samples was determined by Bradford assay. A total of 10 µg of proteins was combined with 4× Laemmli buffer (0.5 M TrisHCl pH 6.8, 10% sodium dodecyl sulfate, 20% glycerol, β-mercaptoethanol, and 0.1% bromophenol blue) and boiled for 5 min.

After that, 12% SDS/polyacrylamide gel was used to separate proteins that ware then transferred onto a polyvinylidene fluoride (PVDF) membrane using the Trans-Blot Turbo Transfer System (BIO-RAD Laboratories, Hercules, CA, USA). The PVDF was probed with primary antibody Immunoglobulin A (IgA) (sc-166334), provided by Santa Cruz Biotechnology (Santa Cruz Biotechnology, Santa Cruz, CA, USA). The primary antibody was diluted 1:1000 in 2% milk and incubated overnight at 4 °C. An enhanced chemiluminescence (ECL) detection system (GE Healthcare, Chicago, IL, USA) and Amersham Imager 600 (GE Healthcare, IL, USA) were used to detect the bands after incubation with horseradish peroxidase (HRP)-conjugated anti-mouse IgG (1:10,000) (Santa Cruz Laboratories, Santa Cruz, CA, USA). The PVDF membrane was stained with Coomassie brilliant blue R-250 and the total protein intensities were used to normalize the intensity of the immuno-stained bands. The blot was analyzed using the Amersham Imager 600 software (version 1.2.0).

### 2.6. Gas Chromatography–Mass Spectrometry (GC–MS) Analysis of Plasma Samples

Metabolite analyses were performed on each plasma samples by Gas Chromatography–Mass Spectrometry using the selected ion monitoring (SIM) mode MS, as reported by Luti et al. [24], with slight modification. Briefly, 25 µL of plasma were diluted with 25 µL of sterile physiologic solution (0.9% NaCl), and metabolite extraction was performed by adding 50 µL of cooled methanol containing 1 μg/mL norvaline (Cat #53721) as the internal standard and 50 µL of cooled chloroform. The obtained mixture was agitated at 1500 rpm at 4 °C for 15 min and phase separation was achieved by centrifugation at 4 °C. The methanol–water phase containing polar metabolites was separated and dried using a vacuum concentrator. Dried polar metabolites were dissolved in 10 μL of 40 mg/mL methoxamine hydrochloride in pyridine (Pierce, ThermoFisher Scientific, Waltham, MA, USA) and kept at 37 °C for 90 min. After dissolution and reaction, 50 μL of MTBSTFA (Cat #375934) was added to the samples that were then incubated at 60 °C for 30 min. Data acquisition was performed by the Intuvo 9000 GC/5977B MS System (Agilent Technologies, Santa Clara, CA, USA) equipped with an HP-5MS capillary column (30 m × 0.25 mm × 0.25 μm). A measure of 1 μL of each sample was injected in split or splitless mode using an inlet liner temperature of 240 °C. GC runs were performed with helium as the carrier gas at 1 mL/min. The GC oven temperature ramp was from 70 °C to 280 °C. The temperature of 70 °C was held for 2 min. Then, the first temperature ramp was from 70 °C to 140 °C at 3 °C/min. The second ramp was from 140 °C to 150 °C at 1 °C/min. The third temperature ramp was from 150 °C to 280 °C at 3 °C/min. Metabolite measurements were conducted using electron impact ionization at 70 eV in SIM mode. The ion source and transfer line temperatures were maintained at 230 °C and 290 °C, respectively. Data analysis was performed using MS Quantitative Analysis software (Agilent, version 10.2). Relative metabolite abundances were determined by normalizing the integrated signal of selected ions for each metabolite to the signal of norvaline and the protein content.

### 2.7. Statistical Analysis

Data are presented as means ± standard deviation (SD) from the seven subjects included in the study. Significance was defined as *p*-value < 0.05 and statistical analysis was performed by *t*-test using Graphpad Prism 8. As measure of the effect size, we reported the partial eta-squared (η_p_^2^), considering the following intervals: η_p_^2^ ≤ 0.01 small effect; η_p_^2^ ≤ 0.06 moderate effect; and η_p_^2^ ≤ 0.14 high effect [25,26].

## 3. Results

### 3.1. Participants’ Characteristics

The match analyzed in the study is an official competition of the male professional team ACF Fiorentina carried out during the Italian championship “Serie A”. A total of 15 players participated in the game which lasted for ninety-seven minutes.

In our study, we considered athletes that had played for at least 60 min; therefore, it comprised a total of seven, including two central defenders (CD), two side defenders (SD), two midfielders (MF) and a striker (ST). The goalkeeper was not considered. The characteristics of the seven professional soccer players are reported in Table 1. In summary, the means of age, height, weight, and BMI were 27.7 years, 174.5 cm, 71 kg, and 23.5 kg/m^2^, respectively.

### 3.2. GPS Analysis

We examined the athletic performance of the seven players during the match by a GPS analysis, considering the maximum intensity of TD, SR, acceleration, and MP as reference parameters describing the intensity of the match.

To estimate the general intensity of the match, we compared the obtained performance values with the data averages during the Italian Serie A Championship 2023–2024, recorded with the same GPS devices from K-sport. Obtained data are reported in Table 2.

Comparing the GPS parameters, the analyzed match showed a metabolic power similar to the average of the Italian championship, with only a 0.7% difference (10.57 and 10.50 W/Kg). However, there were some differences in total distance traveled (−8.6%) and accelerations (−22.4%), which were lower in the analyzed match in comparison to Italian Serie A average. Still, the sprint distance percentage was higher (+17.6%).

Analyzing the selected subjects by department, we showed that there were few differences between the roles (Table 3). The data averages by department is useful to have a general overview of the match dynamics and the competitive level of the opposing team. Side defenders were the players who had the higher MP (+9.5%) and SR (+49.8%) in comparison to the mean of the athletes, while the midfielders had the lower values (MP: −8%; SR: −44.3%).

### 3.3. Plasma Oxidative Stress Analysis

**Figure 1:**
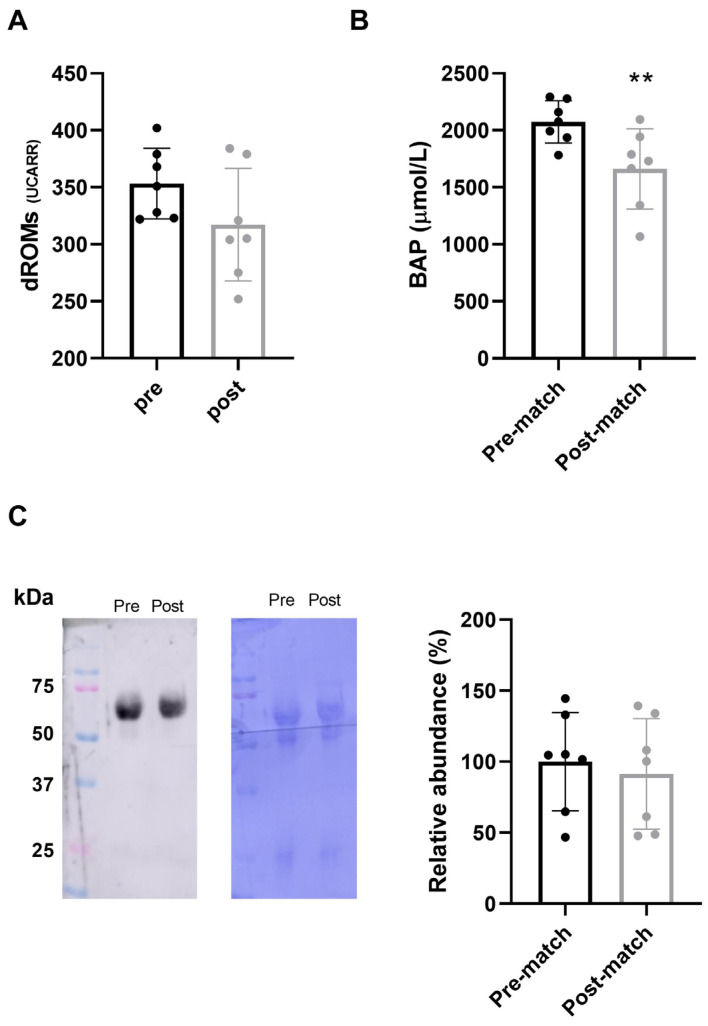
Plasma oxidative stress 24 h pre and 48 h post-match. (**A**) levels of reactive oxygen metabolites using the d-ROMs test (diacron-Reactive Oxygen Metabolites) and (**B**) the total antioxidant capacity evaluated using the BAP Test (Biological Antioxidant Potential) using a free radical analyzer. (**C**) Representative western blot image of protein carbonylation in plasma samples of the same athlete pre and post single play match. Bars show the mean ± SD of the selected players. The statistical analysis was carried out by *t*-test using Graphpad Prism 8 (** *p* < 0.01).

To evaluate the oxidative status of the athletes after 48 h of a “Serie A” match, we measured the antioxidant capacity (BAP) and the levels of oxidative species (d-ROM) in the pre-match and post-match plasma of the seven selected athletes who had played for at least 60 min. The results obtained are reported in Figure 1. We noticed that there was not a statistically significant difference in d-ROMs levels pre- and post-match (Figure 1A, 90%; *p* = 0.162), indicating that at 48 h post-match, players have the same oxidative stress value of 24 h pre-match. At the same time, the BAP levels were reduced post-match in comparison to pre-match (Figure 1B, 80%; *p* = 0.006; η_p_^2^ = 0.745), implying their consumption following the match.

To confirm the absence of oxidative stress 48 h post-match, we evaluated the total plasma carbonylation, which is a well-documented marker of protein oxidation. The results, reported in Figure 1C, indicated that the levels of protein carbonylation pre- and post-match were comparable with a slight difference and were not statistically significant (post-match 90.6%; *p* = 0.414). These data agreed with d-ROMs analysis, confirming the absence of oxidative stress at 48 h post-match.

### 3.4. IgA Levels in Saliva Samples

**Figure 2:**
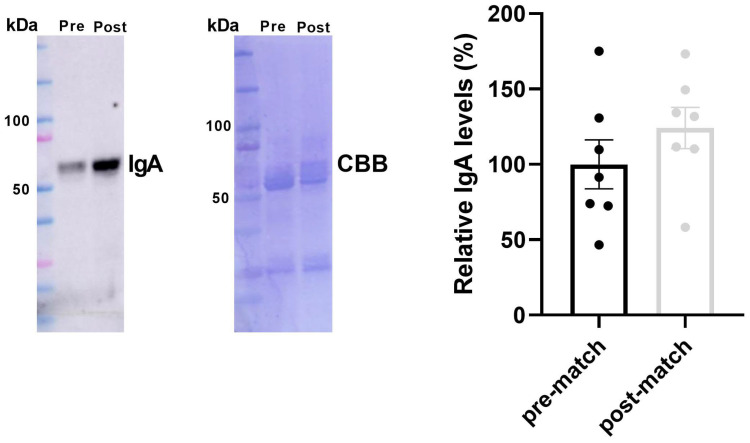
Salivary IgA levels at 24 h pre and 48 h post-match. Representative immunoblot image and relative quantification using the Coomassie brilliant blue-stained membrane (CBB) of IgA in salivary samples pre- and post-match. St: BioRad Precision Plus Protein™ standards.

As a parameter of the immune state of athletes, we analyzed the IgA levels in saliva samples. Saliva collection was performed 24 h pre and 48 h post-match and IgA was assayed by western blot. The results, reported in Figure 2, indicated that the levels of IgA tend to increase post-match in comparison to pre-match, even if in a not significant manner (post-match 124%; *p* = 0.316). Moreover, we found a great subject variability in IgA levels and this is probably the cause of the lack of significant variations being found [27].

### 3.5. Metabolomic Analysis Using Gas Chromatography/Mass Spectrometry (GC/MS)

**Figure 3:**
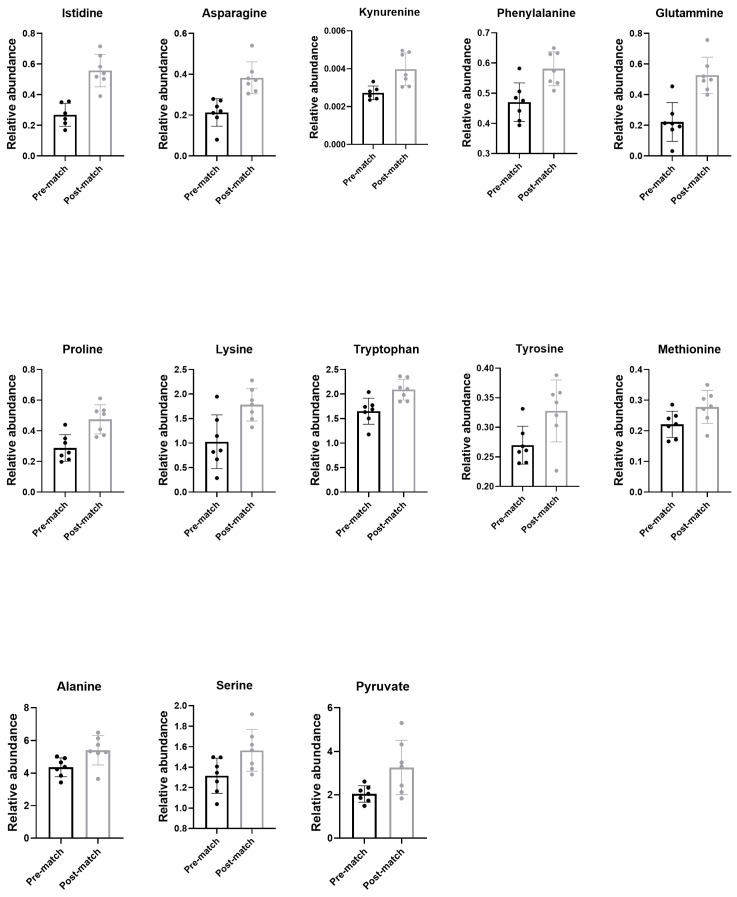
Metabolites identified in plasma 24 h pre and 48 h post-match. Relative abundance of compounds identified by metabolomic GC–MS analysis that changes after the match in the seven players selected for this study.

The pre-match and post-match metabolic profile of plasma from athletes was characterized through GC–MS, as described in the Methods section. The list of the 26 identified compounds and their relative abundance is reported in Table 4. They belong to different metabolic pathways such as glycolysis, Krebs cycle, amino acids, and lipid metabolism. A total of 13 compounds were significantly increased post-match in comparison to pre- match (Figure 3): histidine (208%; *p* < 0.001; η_p_^2^ = 0.800), asparagine (179%; *p* < 0.001; η_p_^2^ = 0.805), kynurenine (146%; *p* = 0.007; η_p_^2^ = 0.586), phenylalanine (124%; *p* = 0.005; η_p_^2^ = 0.751), glutamine (237%; *p* < 0.001; η_p_^2^ = 0.851), proline (166%; *p* = 0.002; η_p_^2^ = 0.675), lysine (174%; *p* = 0.009; η_p_^2^ = 0.568), tryptophan (127%; *p* = 0.005; η_p_^2^ = 0.562), tyrosine (121%; *p* = 0.028; η_p_^2^ = 0.523), methionine (126%; *p* = 0.049; η_p_^2^ = 0.476), alanine (124%; *p* = 0.024; η_p_^2^ = 0.626), serine (119%; *p* = 0.030; η_p_^2^ = 0.731), and pyruvate (160%; *p* = 0.029; η_p_^2^ = 0.621). Interestingly, they were mainly amino acids. On the contrary, the metabolites that had not changed between pre- and post-match are reported in Figure S1. They were Krebs cycle intermediates (α-ketoglutarate, citrate, fumarate, malate and succinate), lipids (palmitic acid and stearic acid), lactate, and some amino acids (leucine, valine, aspartate, glycine, glutamate, threonine and isoleucine).

### 3.6. Metabolomic Interaction Network

Using the list of differentially abundant metabolites identified through GC–MS analysis, an interaction network was constructed to explore relationships between metabolites and genes. This was achieved using MetScape (http://metscape.med.umich.edu (accessed on 13 September 2024)), a Cytoscape plugin (https://cytoscape.org/) that provides a bioinformatics framework for interpreting and visualizing human metabolomics data. MetScape analyzes metabolic networks using an integrated database combining data from KEGG (Kyoto Encyclopedia of Genes and Genomes) and EHMN (Edinburgh Human Metabolic Network). This tool facilitates the identification of enriched pathways from expression profiling data, the construction and analysis of gene-metabolite interaction networks, and the visualization of changes in gene/metabolite datasets.

**Figure 4:**
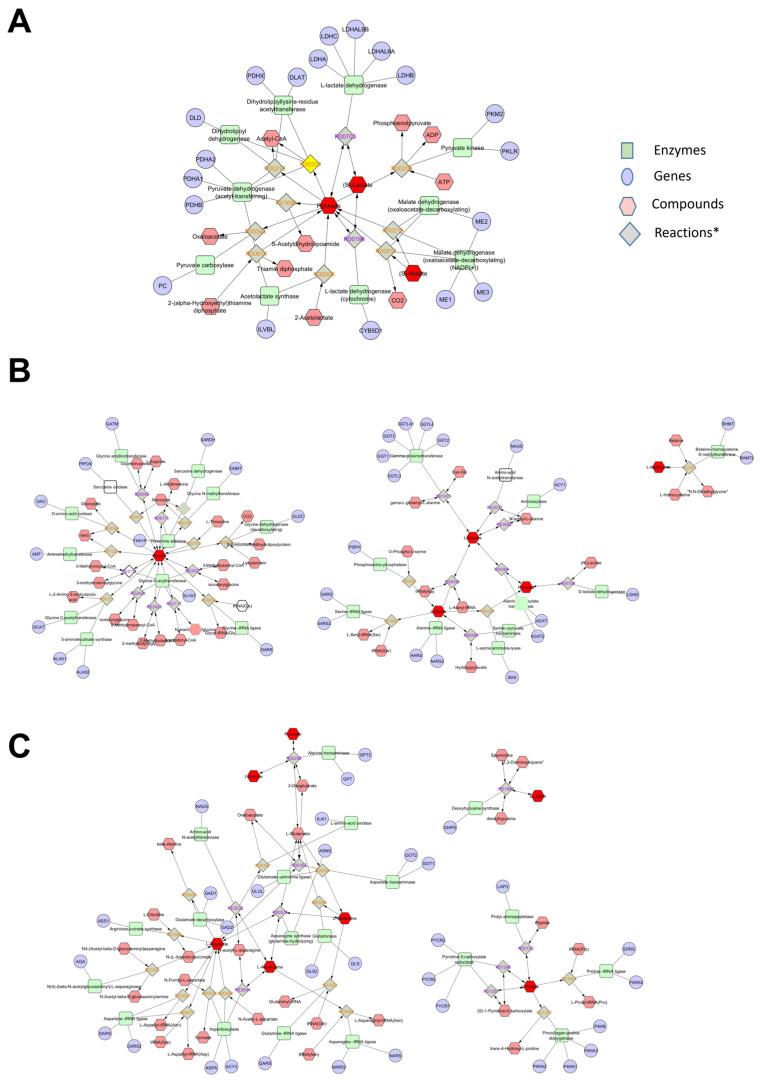
Metabolomics network interaction. Network analysis on plasma metabolites identified by GC–MS (significantly different metabolites on Table 4) was performed using the MetScape 3 App for Cytoscape (http://metscape.med.umich.edu (accessed on 13 September 2024)). (**A**) Glucose metabolism and gluconeogenesis; (**B**) glycine, serine, alanine, and threonine metabolism; (**C**) urea cycle and metabolism of arginine, proline, glutamate, and asparagine. * Each different reaction is reported in the Supplementary Materials.

The input list was generated using only the significant metabolites reported in Table 4. The pathways implicated by the metabolomics analysis are depicted in Figure 4, highlighting the involvement of glucose metabolism, the urea cycle, and several amino acid metabolic pathways.

## 4. Discussion and Conclusions

Football players are subjected to long-term impact during the training period, which can influence physical recovery. By understanding and effectively integrating acute exercise sessions such as match-play during a training period, athletes can maximize their performance and minimize the risk of injury and overtraining [5,28].

The aim of this study was to evaluate the technical and physical profile of a professional football team at the end of a single match during the training period and to correlate it with a metabolic and redox profile. The ACF Fiorentina team used the 4-2-3-1 formation for this single match and the GPS parameters showed that the match-play analyzed generated results comparable to the average of the Italian Serie A championship, especially when regarding metabolic power, which indicates a mere 0.7% difference. These values indicate that this specific match can be taken as an example of a typical football match of professional athletes in order to evaluate the physical and technical performances and compare them with the biological parameters.

In our match analysis, side defenders are the players who traveled the most distance, also having higher values for sprint running distance. Moreover, they also have higher metabolic power values. Overall, these parameters underscore the level of the opposing team, especially engaging the side defenders, particularly in the distance for sprint running. The opposing team, of good technical level, certainly expressed an offensive soccer strategy, engaging beyond average parameters; remarkably, in the defensive department, the central defenders indeed have higher values for acceleration. When a department shows high values of sprint running (SR) distance and acceleration values, it is plausible that many moments of “non-possession” occurred, remaining in negative transition for a long time [20,21,29,30].

The redox analysis highlighted that in our elite athletes, the plasma antioxidant capacity 48 h from the match-play is preserved from the oxidative stress due to the game. Specifically, the BAP levels were significantly lower post-match compared to pre-match, indicating a physiological response to mitigate oxidative stress. This finding is supported by the d-ROM levels, an assay that measures the oxidative stress of blood samples by evaluating the level of reactive oxygen metabolites [31,32], which remained comparable between pre- and post-match samples. The absence of oxidative stress is also evident by plasma protein carbonylation, an index of oxidative stress. We found that there are no significant differences between the levels of protein carbonylation pre- and post-match. It is well known that the quantification of protein damage is one of the most common features of oxidative stress, and protein carbonylation has been often used as a marker for identification of this stress [33,34,35]. In a previous paper, we performed an overview of plasma protein oxidation after exercise, identifying proteins that are targets for oxidation after exercise [36]. This result is further supported by the reactive oxygen metabolite data, which confirmed that an acute stressful event, such as a match, in trained subjects does not modify their level of oxidative stress after 48 h.

Physiological stress in football has often been detected using some salivary biomarkers, although their relationship with physical performance is not yet fully clarified [37]. While vigorous exercise is not good for the immune system, moderate exercise is thought to be protective. A study by Nieman et al. [38] showed that moderate exercise is associated with high natural killer cell activity after six weeks. However, few studies have examined the effect of exercise mode on the rate of salivary-IgA secretion among the same subjects; therefore, we aimed to elucidate changes in salivary-IgA secretion in response to acute exercise (a single match) during the training period. Our results showed that IgA levels at 24 h pre-match and 48 h post-match were comparable, with no statistically significant differences. This finding aligns with the study by Moreira et al. [39], which reported no significant changes in IgA levels before and after a 70 min match. Similarly, Kock et al. [40] observed comparable results when analyzing saliva samples taken pre- and post-competitive rugby matches, as did Morgans et al. [41] when observing 24 h pre and 48 h post-competitive match play of the Elite Russian Premier League. While this study suggests that acute intense exercise does not appear to reduce salivary IgA levels in elite soccer players, this does not imply that IgA should be disregarded as a potential biomarker of inflammation, particularly during periods of overreaching or overtraining [37].

Metabolomics analysis revealed an increase in the concentration of several plasma amino acids and the involvement of glucose and urea cycle metabolism. This is in line with the literature because during exercise, protein synthesis in skeletal muscle decreases and protein degradation increases [42,43]. Moreover, there is evidence that basal amino acid concentrations in plasma and muscle may be higher in trained individuals than in untrained individuals [43]. The percentage of energy supply through the catabolism of amino acids is high during exercise and this is explained by the increase in metabolic processes induced by exercise, in which carbons derived from amino acids are used.

In our athletes, we found an increase in tryptophan. Some authors studied plasma BCAA and tryptophan levels after exercise, demonstrating that plasma BCAA levels remained unchanged over a period while free tryptophan levels were significantly increased after exercise [44,45]. It is known that a reduction in Trp plasma level could be related to muscle fatigue after intense physical exercise [46]. Interestingly, our athletes, who play a match during the training period, show a contemporary increase in kynurenine, one of the tryptophan metabolites. The kynurenine pathway has been identified as playing a critical role in generating cellular energy, producing nicotinamide adenine dinucleotide (NAD^+^) [47]. Therefore, an increase in plasma levels of both these metabolites, Trp and kynurenine, suggest intense mitochondrial activity for energy production [48].

We also found an increase in plasma glutamine level, a well-studied amino acid in relation to exercise. It is a non-essential amino acid and is the most abundant amino acid in plasma. Moreover, skeletal muscle is the main organ involved in its production and release into the bloodstream and several authors reported its decrease in association to physical stress and “overtraining syndrome” [49,50,51].

Moreover, we found an increase in the concentration of plasma alanine, which has a central role in energy metabolism during exercise through its role in the glucose–alanine cycle present in hepatic gluconeogenesis [52,53]. Furthermore, some studies have highlighted that this amino acid decreases when physical effort is prolonged, and its decrease could be predictive of overreaching due to an increase in gluconeogenic demand [54].

Asparagine and aspartate, which we found increased in the post-match plasma of athletes, are precursors of oxaloacetic acid which is a fundamental intermediate in mitochondrial energy metabolism. For this reason, some authors suggest that these amino acids determine a saving of muscle glycogen during intense physical exercise [55,56,57]. The results reported in the literature, which are however controversial, seem to suggest that oxaloacetate may be important in determining the time to exhaustion during prolonged exercise. Overall, these results suggest that an increase in alanine, asparagine, and aspartate plasma levels, as well as the other amino acids we found, indicate a good level of training that is able to prevent physical and physiological symptoms of overreaching.

The primary limitation of this study is the sample size. We selected participants from the same Italian Serie A team, all of whom underwent identical training sessions and recovery strategies. These factors, which could potentially influence the results, cannot be standardized if players from different teams were included. Additionally, one of the inclusion criteria was that players had to have participated in at least 60 min of the selected match, further reducing the number of participants. Therefore, this study should be considered a pilot investigation. Moreover, the data presented pertains to a single match; future research should aim to compare data across multiple matches. In conclusion, in well-trained athletes which performed an acute event such as a single-play match with a metabolic and physical request on average with the Italian championship “Seria A”, at 48 h post-match there is an absence of oxidative stress to the detriment of the antioxidant potential and an increase in several metabolites, which plays a critical role in mitochondrial energy production. Integrating GPS technology with metabolic analysis provides a powerful tool for optimizing player performance, improving tactical decisions, and enhancing overall team success. It represents a fusion of physical performance data with biological insights, paving the way for more scientific and data-driven approaches in soccer.

## Figures and Tables

**Table 1:** Participants’ characteristics.

	Age (Year)	Height (cm)	Weight (kg)	BMI (kg/m^2^)
CD 1	27	182	78.1	23.6
CD 2	24	186	76.6	22.1
SD 1	32	179	71	22.8
SD 2	23	174	64.1	21.2
MF 1	34	179	74.5	23.3
MF 2	31	179	84.9	26.5
ST 1	23	174.5	75.8	24.9
Mean	27.7	174.5	71	23.5

CD—central defender; SD—side defender; MF—midfielder; ST—striker.

**Table 2:** GPS data of the analyzed match in comparison to the averages of the Italian league Serie A season 2023–2024.

	Total Distance (m)	Sprint Running Distance > 25 km/h (m)	Distance Acceleration > 2 m/s^2^	Metabolic Power (W/Kg)
Analyzed match	10,113.4 ± 178.32	283.38 ± 132.80	176.84 ± 33.50	10.57 ± 0.85
Serie A mean	11,070.4 ± 188.20	241.05 ± 21.90	228.00 ± 7.00	10.50 ± 0.20

**Table 3:** GPS data of the analyzed match dividing the players by department.

	Time Played (Minutes)	Total Distance (m)	Sprint Running Distance > 25 km/h (m)	Distance Acceleration > 2 m/s^2^	Metabolic Power
CD 1	97.53	11,398.03	426.71	215.26	10.85
CD 2	97.53	10,557.39	285.7	172.27	10.02
Mean CD	97.53	10,977.7 ± 594.42	356.2 ± 99.7	193.7 ± 30.4	10.6 ± 0.7
SD 1	97.53	12,279.01	335.81	122	11.76
SD 2	91.23	10,998.58	513.44	193.03	11.34
Mean SD	94.38	11,638.8 ± 905.4	424.62 ± 125.6	157.5 ± 50.22	11.5 ± 0.29
MF 1	97.53	10,489.21	161.79	198.8	10.05
MF 2	85.13	8621.21	154.46	141.44	9.28
Mean MF	91.33	9555.2 ± 660.4	158.12 ± 5.1	170.12 ± 40.5	9.66 ± 0.54
ST 1	85	9993.88	201.98	210.57	11.25

CD—central defender; SD—side defender; MF—midfielder; ST—striker.

**Table 4:** Metabolites analysis of plasma players 24 h pre-match and 48 h post-match identified by Gas Chromatography–Mass Spectrometry (GC–MS) analysis.

Metabolite Name	CAS Number ^◘^	KEGG ID °	Mean-Pre	Mean-Post	Difference ^+^	*p*-Value ^Δ^
L-alanine	56-41-7	C00041	4.361	5.414	1.053	0.023989 *
L-asparagine	70-47-3	C00152	0.2131	0.3830	0.1699	0.000937 *
L-aspartic acid	56-84-8	C00049	0.06905	0.09054	0.02149	0.082809 *
L-phenylalanine	63-91-2	C00079	0.4704	0.5812	0.1107	0.004835 *
L-glycine	56-40-6	C00037	3.056	3.292	0.2359	0.318085
L-glutamic acid	56-86-0	C00025	0.9687	1.212	0.2434	0.138978
L-glutamine	56-85-9	C00064	0.2220	0.5267	0.3047	0.000553 *
L-isoleucine	73-32-5	C00407	1.014	1.149	0.1354	0.468522
L-histidine	26,062-48-6	C00135	0.2685	0.5578	0.2893	0.000168 *
L-leucine	61-90-5	C00123	1.925	2.204	0.2784	0.415897
L-lysine	56-87-1	C00047	1.027	1.783	0.7559	0.008950 *
L-methionine	63-68-3	C00073	0.2213	0.2780	0.05671	0.049442 *
L-proline	147-85-3	C00148	0.2885	0.4748	0.1863	0.002407 *
L-serine	56-45-1	C00065	1.316	1.565	0.2489	0.029811 *
L-tyrosine	200-460-4	C00082	0.2699	0.3278	0.05799	0.028104 *
L-threonine	956-48-9	C00102	0.4877	0.4689	−0.01885	0.712030
L-tryptophan	73-22-3	C00078	1650	2090	0.4400	0.005013 *
L-valine	72-18-4	C00183	3041	3265	0.2241	0.471524
kynurenine	2922-83-0	C00328	0.002727	0.003975	0.001248	0.007081 *
Alpha-ketoglutaric acid	328-50-7	C00026	0.05269	0.05919	0.006503	0.301500
Citric acid	5949-29-1	C00158	1.117	1.317	0.2004	0.211565
Fumarate	110-17-8	C00122	0.02126	0.02053	−0.0007238	0.811405
Malate	149-61-1	C00149	0.03063	0.04747	0.01684	0.201045
Succinate	56-14-4	C00042	0.1381	0.1128	−0.02532	0.107247
Pyruvate	57-60-3	C00022	2.040	3.262	1.221	0.029370 *
L-lactic acid	79-33-4	C00186	21.66	26.49	4.832	0.062270

^◘^ Chemical Abstract Service number. ° KEGG identifier (https://www.genome.jp/kegg/ (accessed on 26 July 2024)). ^+^ Difference between metabolite level pre- and post-match. ^Δ^ *p*-value was determined by *t*-test (* *p* < 0.05).

## Data Availability

Data is contained within the article and Supplementary Materials.

## Supplementary Materials

The following supporting information can be downloaded at: https://www.mdpi.com/article/10.3390/antiox14010073/s1, Figure S1: Metabolites identified in plasma 24 h pre and 48 h post-match that does not change, List of reactions emerged from metabolomics network analysis.
