# Supplementary material for: Molecular, Physical, and Technical Performance Response After a Competitive Match in Male Professional Soccer Players
Source: Antioxidants (Basel). 2025 Jan 9;14(1):73. doi: 10.3390/antiox14010073 (PMC11763290; doi:10.3390/antiox14010073)
Supplement: Supplementary file 1 [file antioxidants-14-00073-s001.zip › antioxidants-3304111-supplementary.pdf]

Supplementary Materials

List of reaction emerged from metabolomics network analysis

Glycolysis and gluconeogenesis (Figure 4, panel A):

Reactions:

ID: R00006 Equation: 2-Acetolactate+CO2=2 Pyruvate
ID: R01699 Equation: Pyruvate+Lipoamide=S-Acetyldihydrolipoamide+CO2
ID: R00014 Equation: 2-(alpha-Hydroxyethyl)thiamine diphosphate+CO2=Thiamindiphosphate+Pyruvate
ID: R00210 Equation: Pyruvate+CoA+NADP+=Acetyl-CoA+CO2+NADPH
ID: R00216 Equation: (S)-Malate+NADP+=Pyruvate+CO2+NADPH
ID: R00214 Equation: (S)-Malate+NAD+=Pyruvate+CO2+NADH
ID: R00209 Equation: Pyruvate+CoA+NAD+=Acetyl-CoA+CO2+NADH
ID: R00200 Equation: ATP+Pyruvate=ADP+Phosphoenolpyruvate
ID: R00703 Equation: (S)-Lactate+NAD+=Pyruvate+NADH+H+
ID: R00196 Equation: (S)-Lactate+2 Ferricytochrome c=Pyruvate+2 Ferrocytochrome c
ID: R00344 Equation: ATP+Pyruvate+HCO3-=ADP+Orthophosphate+Oxaloacetate

Glycine, serine, alanine and threonine metabolism (Figure 4, panel B)

Reactions:

ID: R02821 Equation: "Betaine+L-Homocysteine=N,N-Dimethylglycine+L-Methionine"
ID: RE1473 Equation: "L-alanine+glutathione = ""g""-L-glutamyl-L-alanine+L-cysteinylglycine"
ID: RE2642 Equation: "H(,2)O+`N`-acetyl-L-alanine = acetate+L-alanine"
ID: RE2031 Equation: acetyl-CoA+L-alanine = CoA+H('+)+`N`-acetyl-L-alanine
ID: R03038 Equation: ATP+L-Alanine+tRNA(Ala)=AMP+Pyrophosphate+L-Alanyl-tRNA
ID: R00369 Equation: L-Alanine+Glyoxylate=Pyruvate+Glycine
ID: R00704 Equation: (R)-Lactate+NAD+=Pyruvate+NADH+H+
ID: R00220 Equation: L-Serine=Pyruvate+NH3
ID: R00585 Equation: L-Serine+Pyruvate=Hydroxypyruvate+L-Alanine
ID: R03662 Equation: ATP+L-Serine+tRNA(Ser)=AMP+Pyrophosphate +L-Seryl-tRNA(Ser)
ID: R00582 Equation: O-Phospho-L-serine+H2O=L-Serine+Orthophosphate

Urea cycle and metabolism of arginine, proline, glutamate, aspartate and asparagine (Figure 4, panel C)

Reactions:

ID: R03652 Equation: ATP+L-Glutamine+tRNA(Gln)=AMP+Pyrophosphate +Glutaminyl-tRNA
ID: R00256 Equation: L-Glutamine+H2O=L-Glutamate+NH3
ID: R00253 Equation: ATP+L-Glutamate+NH3=ADP+Orthophosphate+L-Glutamine
ID: R01954 Equation: ATP+L-Citrulline+L-Aspartate=AMP+Pyrophosphate +N-(L-Arginino)succinate
ID: R03647 Equation: ATP+L-Aspartate+tRNA(Asn)=AMP+Pyrophosphate +L-Aspartyl-tRNA(Asn)
ID: R05577 Equation: tRNA(Asp)+L-Aspartate+ATP=L-Aspartyl-tRNA(Asp) +Pyrophosphate+AMP

ID: R00489 Equation: L-Aspartate=beta-Alanine+CO2
ID: R03421 Equation: N4-(Acetyl-beta-D-glucosaminyl)asparagine+H2O =>N-Acetyl-beta-D-glucosaminylamine+L-Aspartate
ID: R00357 Equation: L-Aspartate+H2O+Oxygen=Oxaloacetate+NH3+H2O2
ID: R00355 Equation: L-Aspartate+2-Oxoglutarate=Oxaloacetate+L-Glutamate
ID: R00578 Equation: ATP+L-Aspartate+L-Glutamine+H2O=AMP+Pyrophosphate +L-Asparagine+L-Glutamate
ID: R00258 Equation: L-Alanine+2-Oxoglutarate=Pyruvate+L-Glutamate
ID: RE2644 Equation: "H(,2)O+`N`-acetyl-L-asparagine = acetate+L-asparagine"
ID: RE2032 Equation: acetyl-CoA+L-asparagine = CoA+H('+)+`N`-acetyl-L-asparagine
ID: R03648 Equation: ATP+L-Asparagine+tRNA(Asn)=AMP+Pyrophosphate +L-Asparaginyl-tRNA(Asn)
ID: R01252 Equation: L-Proline+2-Oxoglutarate+Oxygen=trans-4-Hydroxy-L-proline +Succinate+CO2
ID: R00135 Equation: Peptide+H2O=L-Proline+Peptide
ID: R03661 Equation: ATP+L-Proline+tRNA(Pro)=AMP+Pyrophosphate +L-Prolyl-tRNA(Pro)
ID: R01248 Equation: L-Proline+NAD+=(S)-1-Pyrroline-5-carboxylate+NADH+H+
ID: R01251 Equation: L-Proline+NADP+=(S)-1-Pyrroline-5-carboxylate+NADPH+H+

ID: RE1899 Equation: "L-lysine+spermidine = 1,3-diaminopropane+deoxyhypusine"

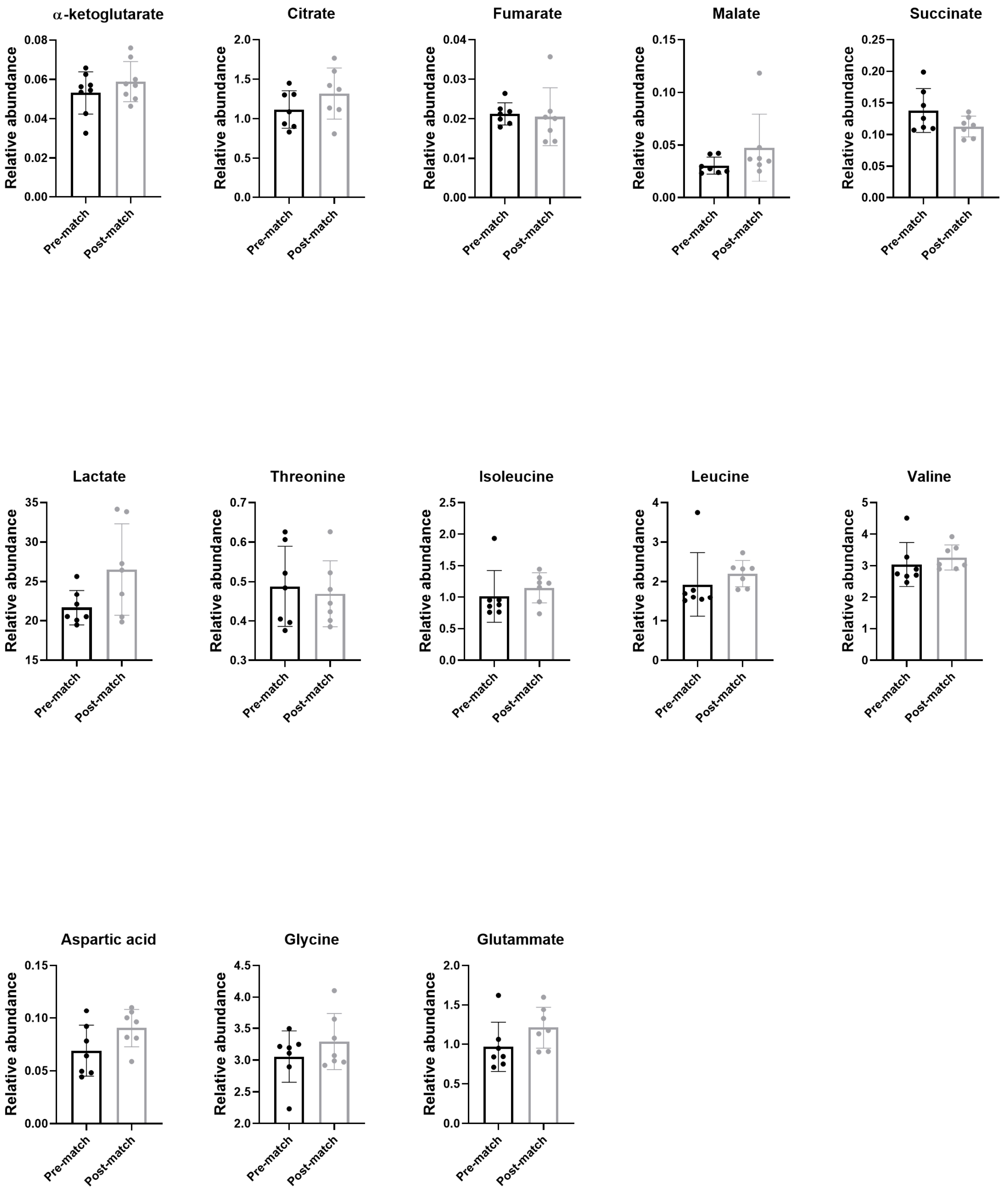

Figure S1. Metabolites identified in plasma 24 h pre and 48 h post-match that does not change, List of reactions emerged from metabolomics network analysis.
